# Integrating Data Directly into Publications with Augmented Reality and Web-Based Technologies – Schol-AR

**DOI:** 10.1038/s41597-022-01426-y

**Published:** 2022-06-14

**Authors:** Tyler Ard, Michael S. Bienkowski, Sook-Lei Liew, Farshid Sepehrband, Lirong Yan, Arthur W. Toga

**Affiliations:** 1 USC Stevens Neuroimaging and Informatics Institute, Keck School of Medicine of University of Southern California, Los Angeles, CA 90033 USA; 2 Zilkha Neurogenetic Institute, Keck School of Medicine of University of Southern California, Los Angeles, CA 90033 USA; 3 Chan Division of Occupational Science and Occupational Therapy, University of Southern California, Los Angeles, CA 90033 USA

**Keywords:** Publishing, Research data

## Abstract

Scientific research has become highly intertwined with digital information, however scientific publication remains based on the static text and figures of principal articles. This discrepancy constrains complex scientific data into 2D static figures, hindering our ability to effectively exchange the complex and extensive information that underlies modern research. Here, we demonstrate how the viewing of digital data can be directly integrated into the existing publication system through both web based and augmented reality (AR) technologies. We additionally provide a framework that makes these capabilities available to the scientific community. Ultimately, augmenting articles with data can modernize scientific communication by bridging the gap between the digital basis of present-day research and the natural limitations of printable articles.

## Background & Summary

Scientific publication is now conducted largely online and distributed digitally, however the fundamental structure of the research article remains a simple document comprised of text and printable figures^1–4^. The stagnation of the scientific article has led to a growing gap between the digital methods employed by modern research and the centuries old standards we use to communicate scientific information. Many efforts have attempted to address this discrepancy^5–18^, however none have yet had the impact necessary to displace the practice of simplifying nearly all forms of data into 2D text and static figures for publication^1–4^.

Inadequate representations of scientific data caused by the natural limitations of printable media are prevalent across the scientific community. As a few cursory examples, three dimensional models are routinely presented from a single angle, time series data as a few snapshots, and volumetric anatomical scans as individual slices. These depictions are often utilized solely for publication as more comprehensive digital representations can be employed throughout the course of scientific research.

Even types of information that can be well-depicted through static imagery are often highly constrained by the limited amount of data that can be feasibly included within an article. For example, using a small printable subset of a larger dataset as a ‘representative’ image is a common publication practice across many disciplines. While this technique is widely accepted, it routinely fails to report the vast majority of data and introduces several considerable complications. The selection of the representative image as well as the degree to which it properly represents the larger dataset can be highly subjective^19^ and vary between authors and readers. In the worst situations a representative image can even be misleading, as an author can purposely avoid reporting problematic sections of their dataset and select only the areas that support their hypothesis.

Of the many efforts that have been made to reconcile modern scientific materials with scientific communication, perhaps the most prominent is the use of supplementary materials to provide online digital media alongside principal articles. Unfortunately, these supplemental materials are rarely viewed by readers. A recent study which measured access to research articles and supplements across three journals (JAMA, JIM, JPED) found median views and downloads of supplements was below 0.04% that of articles^20^. While variation of this rate across additional journals should be expected, an increase in supplementary access by a factor of 1000 would still render these materials unseen by the majority of readers. Other merits of supplementary materials notwithstanding, low access rates indicate they do not impact the way the general readership views scientific material.

Another notable approach to modernise scientific communication can be found in the direct embedding of data into the portable document format (PDF). However, while embedding various forms of media such as 3D objects into PDFs has been available since 2008^15^, the technique remains largely unadopted by the scientific community^4,21^. A recent investigation found that out of 9,705,959 indexed PubMed articles between 2008–2018, 156 had associated 3D PDF data, and only 34 directly included 3D PDF data within the principal article^22^. While the study did not include other forms of PDF embeddable data, such as video, it is indicative that the practice has not been widely adopted into scientific publishing.

Many additional efforts have been made to modernize scientific communication outside of the PDF standard. For example, the Journal of Visualized Experiments^5^ (JOVE) provides a web portal that integrates video directly into published works and Elsevier’s Interactive Plots^6^ enables web-based interactive plots and tables. Wolfram Computational Essays^7^ and Jupyter Books^8^ integrate data and code in a manner that allows readers to interactively manipulate computational communications through web portals or local client programs. Projects like Shiny Apps^9^ and Knitr^10^ also facilitate the creation of HTML/markdown documents and interactive web pages that integrate manipulable data, plots and even 3D information^11^ directly from popular languages like R^23^. Open data repositories such as Figshare^12^ have also begun to implement web portals^13^ that can provide web embedding for video, excel worksheets, text files, and other formats. Additionally, individual journals have created HTML/XML-based format standards such as Elsevier’s Article of the Future^14^, Wiley’s Anywhere Article^15^, and eLife’s Lens formats^16^, which can incorporate various capabilities such as video embedding and improved reference linking.

While some of these attempts to modernize the scientific article have stagnated, others are currently very active projects and their ultimate impact on scientific communication remains to be seen. A comprehensive review of each endeavor to improve the research article is outside the scope of this report, however the sum of every approach has yet to displace the PDF standard and the vast majority of research articles remain comprised of text and static figures on a multipage document^1–4^.

There are many possible reasons the research article has proven so resistant to change. Hesitancy of authors to adopt new techniques that require technical manuscript preparation^4,21^, as well as irregular accessibility of digital materials^24^ have likely contributed. Additionally, the willingness of publishers to implement new technologies^22^, as well as the technical and monetary demands they face while doing so pose additional barriers. Furthermore, the adoption and impact of techniques implemented through a publisher are naturally restricted to that publisher’s domain.

**Fig. 1:**
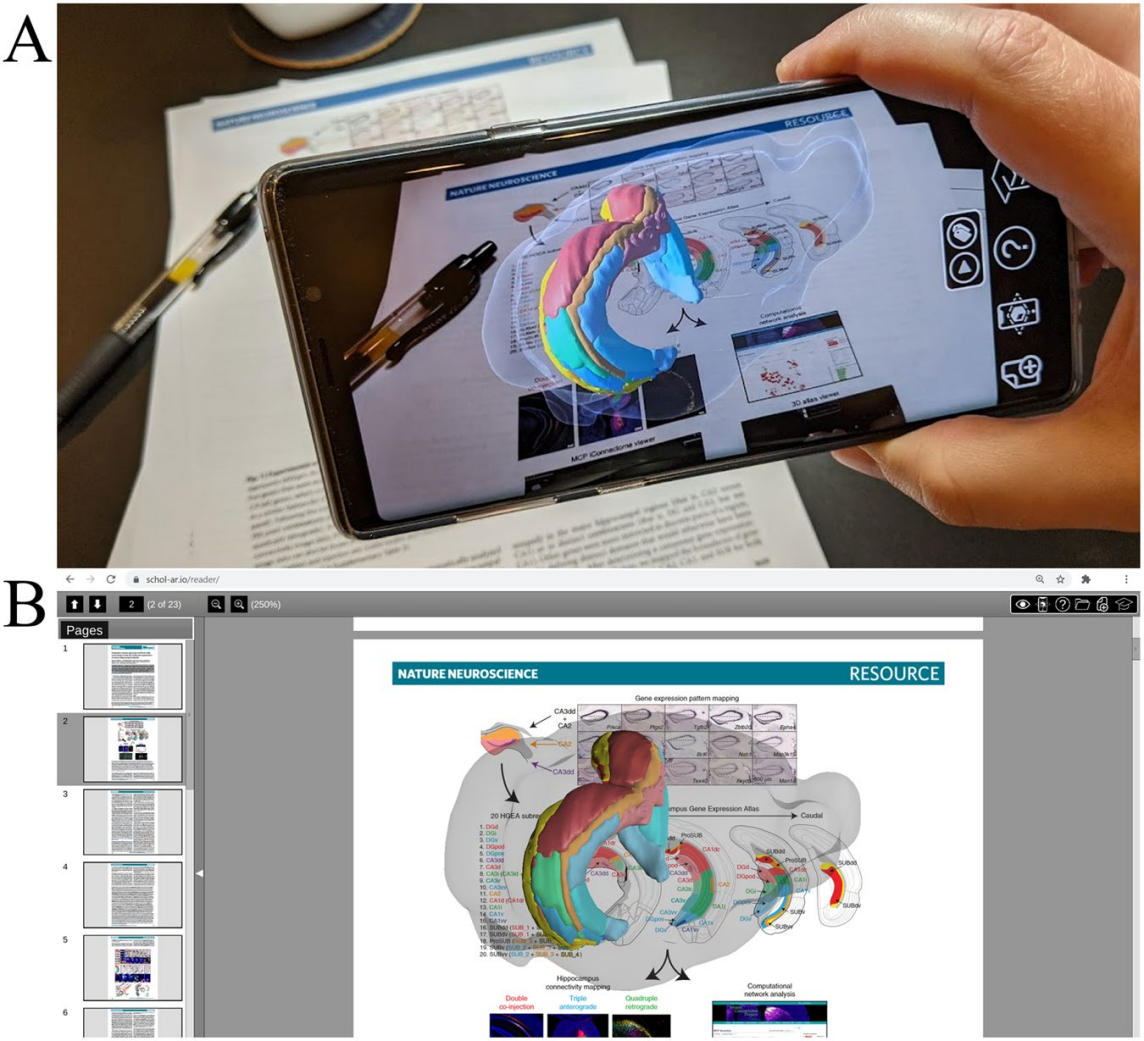
An example of augmented data^46^ directly layered on a publication and viewed through (**A**) an augmented reality mobile application and (**B**) a web-based PDF viewer. This figure is itself augmented and can be viewed through either option (See Results).

Here, we present a unique framework enabling the inclusion of various forms of digital information directly into scientific articles [Fig. 1]. To facilitate adoption, the framework is compatible with standard PDF documents and has been designed to pose minimal burden to authors while maximizing the readership’s accessibility to digital data. Critically, the framework is directly accessible by authors independent from individual publishers and requires no actions or technical capabilities for publishers to implement into their current systems. As such, the capabilities provided through this framework are immediately adoptable across the scientific community.

### Schol-AR

Our framework, termed ‘Schol-AR’, aims to enhance the current publishing system without requiring an overhaul of the system itself. Through this approach standard printable documents can incorporate modern data by layering digital augmentations ‘on top’ of figures [Fig. 1]. These augmentations provide interactive and dynamic viewing of scientific information while requiring no changes to the format of scientific articles or to the publishing system that distributes them. In contrast to many previous approaches, augmenting an article does not require the ability to program, specifically customized data, or technical formats such as XML. Instead, articles remain written as authors are accustomed with the added ability to ‘attach’ scientific data to figures.

Similar to standard scientific figures, augmentations can be independently created by authors and included into manuscripts at any stage of article creation. Augmentations created in this manner can be peer reviewed and validated by publishers as they see fit (see Discussion). The process of creating digital augmentations is designed to be conducted swiftly and is amenable to researchers from a wide variety of backgrounds (see Results).

To facilitate adoption among readers as well as authors, the Schol-AR framework incorporates multiple accessibility options that provide readily viewable data to readers regardless of their preferred medium. Readers who prefer entirely digital modalities can view articles with data displayed directly on top of figures through an online PDF reader. Alternatively, data may be viewed both directly on screens as well as on physically printed medium through a mobile augmented reality (AR) viewer. Furthermore, in addition to research articles these capabilities can also be leveraged to view scientific data on posters, books and other formats.

### Augmented reality & web-based technologies

Separate technologies facilitate data viewing in the mobile and web environments. AR technologies are newer, and typically operate by processing and altering a video stream on a mobile device. AR differs from virtual reality (VR) in that VR obscures your visual world and replaces it with an entirely digital construct, while AR leaves your real-life surroundings viewable and layers digital objects ‘over’ them. For example, the widely popular Pokémon-Go video game layered imaginary game figures on top of real-world locations such as parks and streets, and brought public awareness to AR with over 21 million users in 2016^25^.

Since then, AR has gained considerable attention and applications have been created for many purposes including navigation, art, social networking, marketing and education. While the broad range of AR applications provide a variety of capabilities, those that enable users to create some form of their own AR content are particularly relevant here. Noteworthy examples of such AR content systems include Facebook Spark AR^26^, Snap AR Lens^27^, Vuforia^28^, Wikitude^29^, and Adobe Aero^30^. More specialized content systems can be found in applications such as Artivive^31^ which focuses on augmenting works of art, ARTutor^32^ for augmenting post-secondary educational books^33^, and a platform termed ‘Augment’ tailored towards sales and eCommerce^34^ augmentation.

The possibility of using AR scientifically has also been noted^35^ and researchers have begun investigating possible benefits of utilizing unique types of AR visualizations. For example AR has been used to provide intuitive robotic information^36^, extend and enhance interactive displays^37^, and enable immersive exploration of 3D particle collision information^38^. While these are promising areas of research, here we focus on improving accessibility of digital materials through AR rather than the potential of novel AR visualizations. Other examples employing AR to view well established forms of digital data can be seen in applications such a NASA demonstration of augmented spacecraft^39^, and an application termed BiochemAR which demonstrates the augmentation of a potassium channel^40^.

In addition to specialized applications, researchers have begun creating augmentations inside AR content systems in order to disseminate scientific data. For example, the eCommerce application Augment^34^ has been utilized to view the structure of a bacterial pump^41^. Such employment of AR content systems for scientific augmentation substantially improves the viability of broader adoption by the scientific community by circumventing the considerable barrier of creating individual applications for each instance of scientific augmentation. However, while augmenting literature with some forms of scientific data could be feasibly achieved through several existing AR content systems, it remains an uncommon practice that has yet to gain substantial traction as a medium for scientific communication.

While a myriad of reasons could contribute to the lack of adoption of this technique, many could be attributed to difficulties stemming from the absence of any application or system designed for creating and disseminating scientific augmentations. One such difficulty is that AR content systems do not enable the creation of augmentations directly from forms of data unique to scientific research. The consequences of this should not be overlooked as they have likely played a substantial role in the adoption of other techniques. For example, the direct embedding of 3D data into PDFs appeared to be principally motivated to support the sharing of engineering and design drawings^4^. As such technical circumventions are required to use this technique for various forms of scientific data^4,21^, which is thought to have directly hindered adoption by the scientific community^22^. Furthermore, the integration of augmentations into scientific articles in a manner that enables ready accessibility for all readers can be difficult or impossible to achieve through augmentation systems that are not designed for this purpose. This can not only hamper access through mobile devices, but also constrains viewing to mobile devices. Specifically, augmentations produced through AR content systems are naturally restricted to being disseminated through mobile devices, headsets, or other AR capable hardware. While this limitation does not hinder many of the use cases various AR applications are designed for, it critically excludes access for any readers who are not inclined to use AR capable devices when reading scientific articles.

The framework presented here provides a dedicated system for the augmentation of scientific communication that addresses these concerns. Specifically, the system automatically creates augmentations directly from scientific forms of data and integrates them into scientific publications in an accessible manner. Furthermore, the presented framework is not an AR content system but rather a scientific communication system with an AR component for readers who prefer that medium. As previously discussed, augmentations may additionally be viewed directly on communications through a web-based PDF reader to serve to the considerable portion of the scientific community that prefer articles in this manner.

To facilitate web-based viewing we utilize web graphical technologies, which are more established than AR. Indeed, browser integrated graphical displays have been possible through the web graphics library (WebGL) for many years, resulting in numerous applications of interest to the scientific community. Among them are several non-PDF compatible efforts to modernize scientific communication^8–11^ as discussed earlier, as well as a wide variety of additional applications such as displaying 3D chemical shapes on websites^42^, teaching surgical techniques^43^ and visualizing biological 3D data^44^. While this is only a small sampling of the many scientific WebGL applications, to our knowledge none have previously provided PDF compatible integration of scientific data into articles or other communications.

The novel approach presented here combines both WebGL and AR techniques inside of a framework that enables the hybridization of publications with a variety of digital data formats while maintaining PDF compatibility. Note that as augmentations created through this approach are accessible both through AR and web browsers, the term augmentation is used here to refer to the visualizations themselves and is not specific to AR viewing.

## Results

### Augmented reality augmentation viewing

**Fig. 2:**
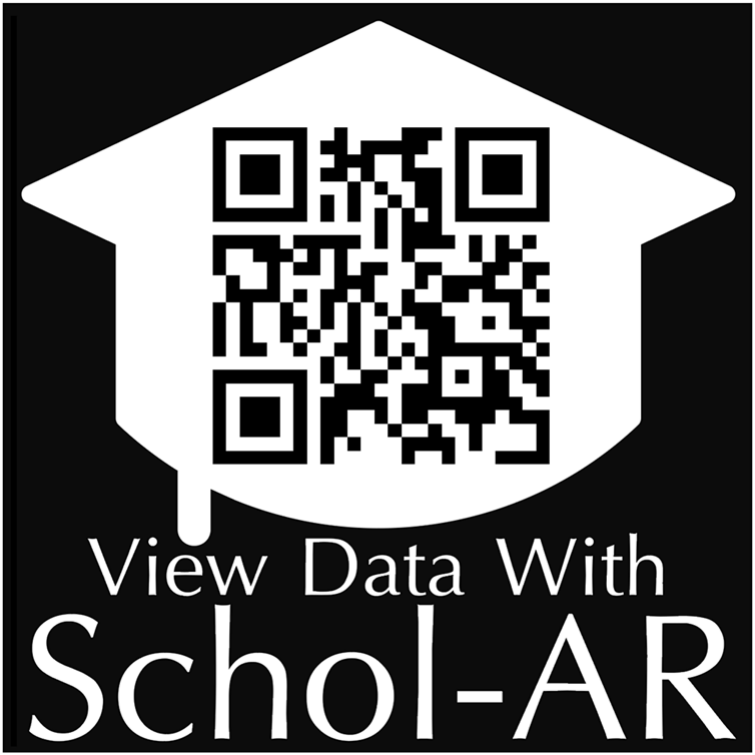
A dual-purpose QR code that: (**A**) When read by the generic camera application or other QR reader directs to Schol-AR app download. (**B**) When read by Schol-AR will download and activate the augmentations of this report.

The augmentations demonstrated in this report, as well as any others created through the system described here are viewable through the Schol-AR application, which is supported on both iOS and Android. Automatic download is available by scanning the QR code in Fig. 2 with a mobile device and following the onscreen prompts. Alternatively, manual download is available through searching the appropriate app store for “Schol-AR.”

Once running, aim the application at the QR code in Fig. 2 in order to load the appropriate augmentations for this publication. After the download is complete the camera may be aimed at any figure, excluding Fig. 2, in order to view accompanying augmentations. As previously mentioned, augmentations may be viewed in this manner on either printed or digital versions of this report.

### Web-based augmentation viewing

The web-based PDF viewer facilitates the viewing of augmentations outside of mobile devices. To view the augmentations in this report through a web browser, navigate to www.Schol-AR.io/reader and open the PDF of this document when prompted. Alternatively, a direct link is available to open both this article and its augmentations (see Methods).

We encourage the reader to view the augmentations of this report through either the web-based or AR application, as next we consider several examples taken from the field of neuroscience that demonstrate how augmentation can facilitate the communication of scientific information.

### Example augmented data – expanded figures & image stacks

Image stacks are a highly prevalent form of data that are typically reduced to a single ‘representative’ slice for publication, as it is impractical to portray entire datasets in print. This practice commonly leaves out over 95% of the data, a limitation that can be directly addressed through augmentation.

**Fig. 3:**
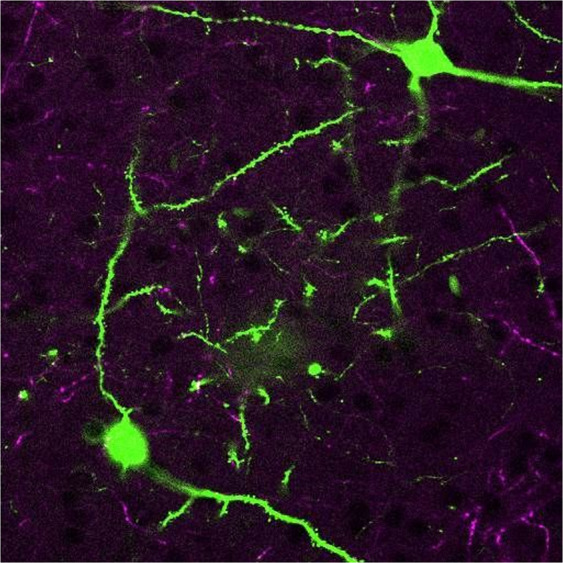
Augmented cellular data from the mouse medial prefrontal cortex^45^. Augmentation enables additional slices to be viewed in sequence via the swipe or click-drag gestures.

To demonstrate, Fig. 3 is a representative image that displays a two-photon microscopy scan of fluorescently labeled mouse neural tissue from the medial prefrontal cortex^45^. When viewed as an augmentation Fig. 3 expands from an individual slice to a comprehensive view of the entire dataset. This approach can be generalized to many additional types of data that do not fit easily into standard images, such as electrical diagrams or large table charts. While these cases clearly benefit from augmenting more data than feasible to print, our remaining examples further benefit from digital representations that could never be printed.

### Example augmented data – 3d models

**Fig. 4:**
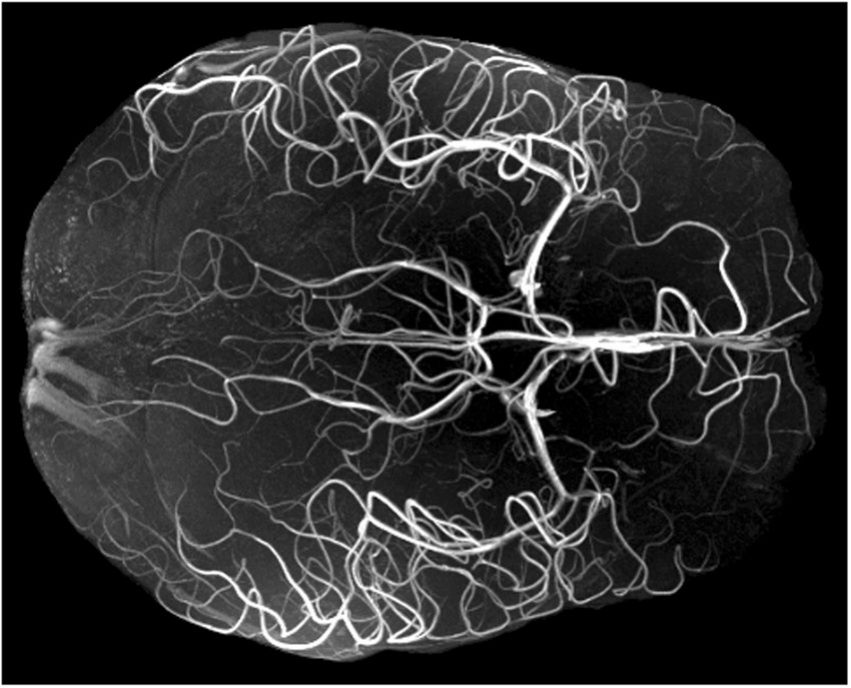
MRI data of the human arterial system. 3D model rotation can be controlled through the web interface and/or mobile device touchscreen.

Many scientific fields utilize three dimensional models for concepts ranging from chemical interactions and molecular activity to the anatomy of cells and larger structures. Regrettably, the limitations of print often result in 3D models being poorly represented or entirely omitted from communications. As an example, Fig. 4 portrays an MRI time-of-flight image which emphasizes the blood flow of a human brain. The figure is a maximum intensity projection (MIP) representing 3D information projected down to two dimensions and is a standard way to communicate this type of data. Viewing the augmented 3D model of this data reveals the extent of simplification imposed by 2D representation as well as how augmentation addresses the issue through comprehensive and interactive depiction.

The augmentation of Fig. 1 further demonstrates 3D model data of genetically defined subdivisions in the hippocampus brain region of rodents^46^. This map is one of the most advanced representations of the hippocampus to date, and benefits from 3D digital representation as well as the dynamic labels and viewing controls that supplement the augmentation.

Ultimately, these augmentations facilitate the communication of 3D structured data, a form of visualization that can be advantageous for a wide variety of concepts and information in addition to those demonstrated here.

### Example augmented data – videos

**Fig. 5:**
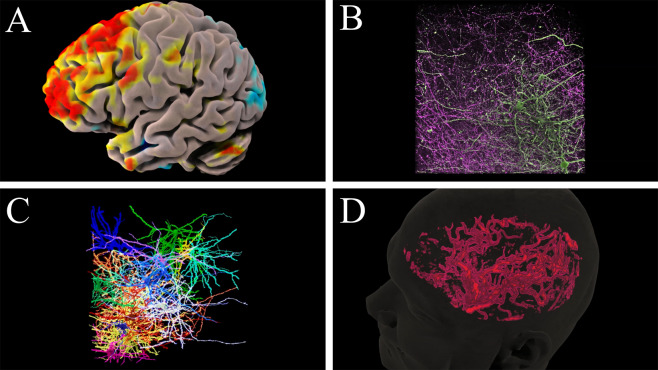
Various MRI and microscopy^45^ datasets. The accompanying video may be paused and restarted from the menu on the web interface or mobile device that appears when viewing this augmentation.

Video media can also be directly augmented onto figures, offering an additional avenue for the inclusion of digital information in communications. Figure 5 briefly demonstrates how the animated nature of video formats can enhance the representation of temporal and spatial data.

Figure 5A depicts the temporal components of fMRI blood oxygenation, which is used to infer the neural activity of brain regions over time. Figure 5D likewise demonstrates the temporal component of arterial blood flow throughout a cardiac cycle. A more comprehensive depiction of these datasets can be viewed through the augmented video that accompanies the static figure. The accompanying video of Fig. 5B introduces a temporal component to the two-photon microscopy dataset from Fig. 3 that better depicts its underlying tangled structure. Figure 5C further utilizes animated augmentation to illustrate the synaptic connectivity of those structures.

These examples are only a small sample of the many forms of data, ideas and concepts that can benefit from video representation. Improved dissemination of this format could facilitate video documentation, demonstrations, and explanations to become less of an exception and more of a standard across the scientific community.

### Augmented data example – volumetric data

**Fig. 6:**
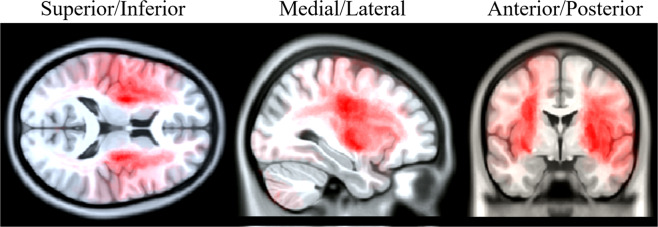
Combined MRI scans of over 200 stroke affected patients, illustrating which regions are most commonly lesioned across patients^47^. The accompanying augmentation enables readers to scan through the entire dataset across thresholds and dimensions.

Volumetric 3D data is a cornerstone of many imaging related disciplines and is typically reduced to representative slices for publication, as shown in Fig. 6. This practice not only fails to portray the vast majority of the dataset, but the reported subset is further constrained to a single statistical and/or numeric threshold. These considerable limitations represent a significant barrier to comprehensively communicating the underlying information.

The augmentation of Fig. 6 demonstrates spatially relevant viewing of an entire volumetric dataset in three dimensions with an adjustable overlay threshold. In this case, the color mapped overlay represents how commonly various neural regions are lesioned across hundreds of stroke subjects^47^, and can be adjusted via a slider interface.

In contrast to the earlier examples which accommodate data from a great many scientific fields, this augmentation technique is limited to fields that utilize three-dimensional volumetric data. We include this example here as the first of its kind, anticipating many more specialized augmentations will be created for specific fields and disciplines that rely on unique forms of data.

### Augmentation creation

Authors may create augmentations from their own data and integrate them into publications through a web interface at www.Schol-AR.io. The process of creating augmentations involves uploading the figure(s) to be augmented and associated digital media – such as video files, 3D models, or any other form of data demonstrated here. Once uploaded, the data is automatically processed and optimized for widespread distribution through both web and mobile viewing. Augmentation creation is independent of AR or web-based viewing, and only needs to be performed once to function across both mediums.

Reader accessibility to augmentations is established through a unique QR code which is automatically provided for each augmented project. It is recommended that authors include this QR code directly with their publication, poster, or other project for ease of access. Including the QR code as a figure in a manuscript PDF also enables the document to be immediately viewable through the web-based viewer. If for any reason the QR code cannot be included in the document – such as when augmenting a previously published report, authors may request their augmentation(s) be manually associated with a specific PDF to enable web-based viewing.

Detailed examples and an updated list of augmentable data formats can be seen at the augmentation creation website.

### Limitations and considerations

This approach is designed to enable broad viewership of scientific digital media, and as such is only appropriate for data that is intended to be widely accessible and viewed. Additionally, scientific data is often unsuitable for rapid downloading and viewing across a wide assortment of mobile devices and browsers. In order to provide these capabilities an automatic re-encoding is performed on uploaded data, which affects it in a variety of ways. These changes should typically be undiscernible by the human eye, however after data is uploaded it should be viewed to verify its fidelity. Furthermore, re-encoded data is often altered in a manner that is visually identical but structurally reformed. As such, this project is not intended for the distribution of data for viewing with other software, further analysis, or any other purposes outside of viewing through the framework. These capabilities can be leveraged through a wide variety of existing services such as Figshare^13^, Mendeley Data^48^, Dryad^49^, IEEE Dataport^50^, Dataverse^51^, or others. Pending community interest, we may provide authors the ability to distribute unprocessed data directly through the framework, however at the time of writing such data should be obtained directly from the authors or their chosen data-sharing platform outside of the augmentation system.

All data associated with this project are processed and stored through reputable cloud service providers and undergo regular backup outside of cloud storage locations. We intend to maintain this system indefinitely, however as with all online resources there is a possibility that the service will discontinue. The most plausible event in which this would occur is if the system is not sufficiently adopted, resulting in low or no activity. If the project is discontinued, viewing and creating augmentations would no longer be available. While we consider this scenario highly unlikely, the depreciation of augmentation services would not interrupt the accessibility or distribution of any augmented articles. This benefit is due to the augmentation technique not requiring technical adaptations to the existing publication system, PDFs or other mediums.

Lastly, the project provides the ability to create, disseminate and view various form of digital visualizations as scientific augmentations, however not all data can or should be viewed in such a manner. For example, in some cases 3D digital models can be highly self-occluding, or suffer from misleading perspectives both inside^52^ and outside^53^ mobile devices. Ultimately, the capability of any visualization to properly communicate the underlying information should be evaluated by the author.

## Discussion

This project combines techniques previously utilized in scientific communication with several novel contributions. As previously discussed there are many tools that facilitate AR augmentation^26–34^ or the enhancement of online scientific digital documents^5–16^. However, to our knowledge this is the first project that enables viewing of data both directly on digital documents and through mobile AR, expanding accessibility to data for readers on their preferred medium. Additionally, this is the first system to provide AR capabilities designed and intended for the communication of scientific research, and as such directly accepts and optimizes the presentation of research-specific types of data.

Furthermore, this is to our knowledge the first attempt to modernize the journal article that does not require publishers to implement new technical capabilities. Even the most undemanding solutions such as digital supplementary materials or PDF data embedding require publishers to store and distribute a substantially larger amount of data and are not supported by all journals. We anticipate that universal accessibility across all scientific journals will facilitate adoption throughout the scientific community. Comprehensive accessibility could prove further beneficial by preventing the divergence of communication standards between larger and more technical journals from smaller journals that cannot feasibly integrate emerging technologies. Furthermore, this style of approach averts the reliance of scientific disciplines on journals that accommodate specific types of data, which could hinder cross-disciplinary innovation.

There are many promising future directions for this project that could further benefit the scientific community. One obvious development we are eager to pursue is expanding the types of data directly accepted into the system. We intend to implement additional support for both general forms of data, such as sound for both data and commentary, as well as many more specialized forms of data that individual scientific disciplines rely on. Additionally, as previously mentioned the project could be adapted to share unprocessed raw data for further analysis in addition to viewing. Furthermore, the digital architecture of the project is also well suited to implement social features that could prove beneficial for scientific communication. For example, the system could provide communication channels between authors and readers, or cross-journal commentary forms to facilitate dialog regarding related articles. These and other features could prove invaluable for promoting communication directly relevant to specific threads of research.

While many of the capabilities discussed here are promising, their specific implementation requires careful consideration before deployment. Fundamentally, we aim to provide capabilities in a manner that can be selectively adopted and further customized by various sections of the community as they deem appropriate. This applies to future possibilities such as social interactions as well as other components like the amending of augmentations after publication. For example, journals may implement a policy that requires authors to transfer the ability to modify an article’s augmentations to the publisher once the article is accepted. With this policy authors could freely augment their articles as they see fit, while publishers could effectively lock an article to its present state at the moment of publication. Alternatively, journals that have more interest in dynamic, ‘live’ content may encourage authors to update augmentations as more data becomes available. Overall, the framework aims to provide infrastructure to support the technical components of scientific communication while leaving control of how these capabilities are implemented in the hands of authors and publishers.

The myriad of possibilities supported through this framework may appear complex, however the actual use of the technique can be very simplistic. One straight-forward example of augmented publishing begins with an author preparing a typical manuscript. As figures are created for the article, augmentations of those figures are also created through the framework presented here. The unique QR code (see Results) is then included as a figure in the manuscript before submitting the article for review. Once submitted in this manner a journal can effectively review the article and augmentations. If the article is accepted, the journal may simply publish the manuscript as a standard PDF and augmentations will automatically be accessible to readers.

Lastly, it should be noted that this project is adaptable across other forms of media as well as through future technologies. For example, there are no barriers to accommodating full digital viewing of other formats in addition to PDF as they become prominent. Data viewing can also be integrated through technologies such as AR glasses and headsets should they gain relevance. Perhaps most importantly, the project is also well suited to enhance other forms of scientific communication such as posters, pamphlets and books. These avenues are not only promising for promoting public outreach and enhancing training, but also for supporting the communication of information at scientific meetings and conferences.

In summary, the modernization of scientific communication holds incredible potential for the research community but has proven historically difficult to achieve. Here we introduce a publisher independent framework that enables the augmenting of digital media onto scientific communications in a highly accessible manner. Ultimately, this project aims to bridge the gap between scientific data and scientific communication, improving our ability to effectively exchange modern concepts, ideas, and information.

## Methods

### Augmentation viewing - AR mobile app

While there are many AR technologies present in modern day mobile devices, we specifically focus on the ability to layer digital media onto static printable images. The basic mechanic this entails is, when a mobile device is aimed at a scientific figure any accompanying digital media such as 3D models, videos, volumes, and interactive displays appear on the mobile device over or around the figure. This is achieved by scanning the mobile camera video stream for specific ‘target figures’, which are the augmented images themselves. Once a target figure is detected digital media associated with that specific figure appears ‘on top’ of the video feed displayed on the mobile device.

Scanning for target figures is a highly optimized technique which involves converting images into scale and rotation invariant representations, then efficiently searching a video feed or other input for them. Throughout development we have alternated through several extensively developed libraries that support many variations of this approach. At time of writing our current implementation utilizes ARCore^54^ and ARKit^55^ for Android and iOS respectively.

Once a target figure has been detected augmented data is displayed ‘on top’ of it through a variety of rendering approaches appropriate for the type of data being displayed. The techniques employed to display video, 3D models, images, and all other augmentations demonstrated here are well documented and outside the scope of this report.

The mobile application additionally utilizes a QR code scanning system. Each augmented article or project is provided with a unique QR code, which is scanned before viewing augmentations. Once an augmented project’s QR code has been detected, the application downloads all relevant target figure representations and augmentations from Schol-AR cloud infrastructure. After downloading completes, the application begins scanning for target figures and will display associated digital media once detected. Overall, the QR system enables the application to selectively download relevant data and scan only for specified target figures instead of the entire database of all possible figures, which would be computationally infeasible.

### Augmentation viewing - web-based PDF viewer

The web-based PDF viewer functions similarly to the AR mobile application, with the notable exception that QR code and image scanning are performed on the document itself in place of a camera feed. At time of writing image scanning in this condition is implemented through the Open Source Computer Vision Library (OpenCV)^56^.

Additionally, in contrast to the mobile viewer which scans for a QR code until one is detected, the web-based viewer only scans for a QR code in the discrete stage of opening a document. In the situation where no QR code is present inside the PDF document, the web-based reader communicates with Schol-AR servers to verify if the PDF itself has been manually associated with an augmentation project per author request (see Results). If such a record is found, loading continues, and the document can be scanned for the appropriately associated target-images. In the situation where a PDF has no QR code or manually associated augmentations, no augmentations will be displayed.

The web-based PDF viewer has the additional ability to open specific PDFs without the having them locally saved on disk. For example, this report can be viewed directly without first downloading this document at www.Schol-AR.io/publications/Ard2022. This type of direct link is highly conducive for accessibility and dissemination of scientific content. However, this capability is currently only available upon request as the legality of hosting each document must be evaluated and/or traffic must be handled in a manner where each user is authenticated by a host who holds the intellectual property rights of the PDF. This is particularly relevant for documents published in journals that are not open-access and may not be an option in all cases. Alternatively, opening a locally saved PDF through www.Schol-AR.io/reader avoids this complication and is available in all cases, as is viewing through the mobile AR viewer.

### Augmentation creation for authors

The augmentation creation system is designed for simplicity and accessibility to accommodate the wide variety of technical backgrounds present across the broad scientific community. The system is implemented as a web site (www.Schol-AR.io) where authors create an account and can augment their individual papers/projects. When augmenting a paper or other communication, authors name their project and upload target figure(s) and associated digital media - such as video file(s) or any other format demonstrated here. Fundamentally, the only additional step necessary for authors is the inclusion of an automatically provided QR code with their document and their augmentations will be immediately accessible through both the mobile and web-based viewers.

While these are commonly the only actions necessary for the successful augmentation of figures, additional options may be used to customize the appearance and functionality of augmentations. For example, the augmentation of Fig. 1 demonstrates 3D labeling and simplistic animations of the anatomical models. These and many other behaviors may be accessed through an ‘edit mode’ in the mobile application that is only available to the creator of the augmentation. This mode allows the simultaneous viewing and editing of augmented content, that once modified will adjust how the augmentation is displayed to all readers. All augmentations may be modified in a variety of ways, such as scale and position relative to the detected target figure. Furthermore, some augmentation types have additional optional modifications, such as the coloring, animation, rotation and labeling of 3D models. Overall, the goal of these customization options is to provide authors the means to adjust their augmented data to display as intended in cases where the default processing is not ideal. More detailed instructions and specific demonstrations can be seen at the augmentation creation website.

It is additionally noteworthy that not all target figures are optimal for detection, and some may entirely fail to be properly detected. As a simplistic example, searching a camera feed or PDF document for a target figure comprised of a single solid line would result in many false positives and ultimately fail to produce the desired behavior. To address this, authors are provided a detectability rating for each uploaded target figure. While many figures are satisfactory for detection without any changes, some with very poor scores will require adjustment. For example, if a figure does not have enough contrast or variance, it may be necessary to add a border or include some of the text surrounding the figure to improve detection. Detailed instructions for improving the detectability of figures can be found at the augmentation creation website.

## Data Availability

Data sharing is not applicable to this article as no datasets were generated or analysed during the current report. Access to datasets from other published works displayed here for demonstrations purposes should be obtained through the associated publication.

## Code availability

This project supports the open accessibility of viewing scientific data, however the project itself utilises licensed code which cannot legally be provided openly. No code from this project generates any scientific results or performs any form of scientific analysis.

The code base consists of several components, namely three clients for Android, iOS, and webGL for viewing and interacting with augmentations. Additional components are integrated through reputable cloud service providers, such as file storage and scalable computation to provide image processing, file ingestion, conversion of uploaded data, and QR creation capabilities. A web server and database handles client requests, serves responses, and enables creation of new augmentations. The components of this project are inter-reliant, and do not function in isolation.

Individual licensed and proprietary components handle many aspects throughout the codebase, including PDF handling and processing (‘PDF Renderer’ by Paroxe), webGL computer vision support (‘OpenCV for Unity’ by Enox Software), animated motion (‘iTween Native Extension’ by Pixelplacement), debug and error reporting (‘Lunar Mobile Console’ by SpaceMadness) and processing of input clicks/gestures (‘Fingers’ by Digital Ruby).

Please contact Tyler.Ard@loni.usc.edu for specific requests or further information.
